# Trial of an Inexpensive Training Simulation Model for Laparoscopic Appendicectomy

**DOI:** 10.7759/cureus.71980

**Published:** 2024-10-21

**Authors:** Anjana S Kumar, Sendhil Rajan, Adeline Rankin, Mina Youssef

**Affiliations:** 1 Norwich School of Medicine, University of East Anglia, Norwich, GBR; 2 General Surgery, Norfolk and Norwich University Hospitals NHS Foundation Trust, Norwich, GBR; 3 Breast Surgery, Norfolk and Norwich University Hospitals NHS Foundation Trust, Norwich, GBR

**Keywords:** core surgical training, foundation doctors, general surgery, healthcare simulation, laparoscopic technique, simulation in medical education, surgical education, surgical skills-based training

## Abstract

Introduction

Laparoscopic surgery is now the gold standard for many common procedures; however, there has been a lack of emphasis on laparoscopic training at the junior level. Simulation is an effective form of training in surgery, but surgical simulation models and courses can be expensive and inaccessible to medical students and foundation doctors. With procedures becoming more minimally invasive, it is key that we train laparoscopic skills at an earlier stage; and to do so, we need to remove the barriers of cost and accessibility. Our study aims to develop and assess the effectiveness of a simulated laparoscopic appendicectomy training model in developing laparoscopic knowledge, skills, and confidence in a cohort of foundation doctors.

Methods

A simulated mesoappendix model was fashioned from supermarket chicken wings with the bone removed, and it was put inside a laparoscopic box trainer with laparoscopic tools and equipment. This was trialed in a surgical-themed hub day for foundation doctors in the East of England Deanery, UK, and the simulated model was set up as a workshop following a teaching session detailing the steps of a laparoscopic appendicectomy. Participants completed questionnaires pre- and post-session to assess perceived skills, experience, and confidence in laparoscopic surgery, laparoscopic skills, and the usefulness of this training model.

Results

A total of 29 foundation doctors with limited formal surgical training completed the survey. The model is quick to prepare and assemble, costing around £0.30 ($0.40). Trainees found the model acceptable and helpful in developing their laparoscopic skills and knowledge of laparoscopic surgery. There was also an increase in self-perceived confidence in performing laparoscopic procedures under supervision.

Discussion

This modification to the model allows the dissection and division of a simulated mesoappendix using only laparoscopic scissors and forceps in a box trainer. This simulation model is a promising and inexpensive tool which can be used for early-stage laparoscopic training for medical students and junior doctors.

## Introduction

Over the past three decades, there has been a major shift from open procedures to minimally invasive procedures, driven by benefits such as lower postoperative pain, shorter hospital stays and recovery periods, as well as better cosmetic outcomes [1,2]. Laparoscopic surgery is the gold standard for many common procedures, including appendicectomies, cholecystectomies, hysterectomies, and even colectomies. Despite this, there are significant barriers to formal laparoscopic training due to cost and accessibility, with formal courses often aimed at surgical trainees. Historically, there has been a lack of emphasis on laparoscopic training at the junior level, specifically foundation trainees and medical students [3]. Laparoscopic appendicectomy is one of the most common emergency general surgical procedures, with around 50,000 performed annually in the UK [4,5]. It is typically one of the first surgical procedures that surgical trainees learn to perform independently and is recognized as a safe operation in developing surgical trainees' laparoscopic surgical skills [1].

Simulation is an effective teaching tool to provide medical students and junior doctors with an introduction to surgical skills in a risk-free environment [6]. Simulated training sessions build familiarity with operative steps and the use of laparoscopic tools and confidence in laparoscopic surgical skills. High-fidelity silicone appendix models and laparoscopic courses can be very expensive; moreover, these synthetic models are not biodegradable and do not accurately represent the feel and nature of handling real tissue.

In response to these challenges, O'Connor and Paraoan described the use of supermarket-sourced chicken wings and gammon in laparoscopic box trainers to simulate laparoscopic appendicectomy with electrosurgical tools [7]. This model offers a more accessible and cost-effective training model for junior surgical trainees; building on this concept, we further simplified this model for beginners to laparoscopic surgery, to simulate the steps of appendicectomy and build familiarity with laparoscopic technique, tools, and equipment, without the use of electrosurgery.

Our study aimed to evaluate if our modified training model is feasible, easy to replicate, and acceptable as an early-stage laparoscopic training model. Additionally, we sought to assess its effectiveness as a training model in developing laparoscopic knowledge, skills, and confidence in a cohort of foundation doctors. 

## Materials and methods

Model

**Figure 1:**
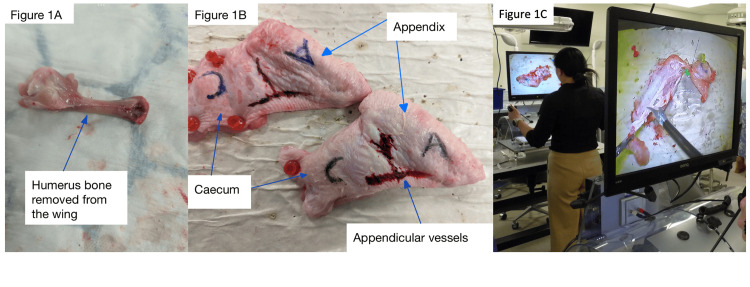
Series of photographs showing the simulated appendix model. (A) (Left) Photograph showing the humerus bone removed from the chicken wing. (B) (Middle) Photograph showing the deboned chicken wing secured to a corkboard, marked with A (appendix), C (cecum), and appendicular vessels drawn on. (C) Laparoscopic tissue photograph showing the dissection on a simulated model in laparoscopic training boxes during the workshop.

Supermarket chicken wings were used to simulate the appendix and cecum. We modified O'Connor and Paraoan's technique [7] to negate the use of energy devices in mesoappendix division and ligation, by removing the humerus bone from the wing to facilitate dissection using laparoscopic dissecting scissors alone (Figure 1A). The wing was secured to a corkboard with pins and a surgical marker used to annotate the wing as "cecum" and "appendix" and to draw on the appendicular vessels. The "fleshy" skin border of the wing represented the "floppy" nature of the mesoappendix (Figure 1B).

Trial

We evaluated the use of this model during a regional Surgical Hub Day designed for foundation doctors (postgraduate years 1 and 2) interested in surgery in the East of England Deanery. 

Prior to the hands-on simulation, a lecture was given to explain the operative steps of a laparoscopic appendicectomy: dissection of the mesoappendix, isolation and division of the appendicular artery, application of Endo-loops, division of the appendix, and retrieval in an Endo-bag, fashioned from a latex glove. This was followed by supervised practice using the chicken wing model in standard laparoscopic box trainers with laparoscopic graspers, forceps, and scissors with two-to-one supervision by an experienced general surgeon, with laparoscopic experience (Figure 1C). Using this method, we were able to use eight laparoscopic box trainers simultaneously.

Participants completed questionnaires pre- and post-session to assess perceived skills, experience, and confidence in laparoscopic surgery and the usefulness of this training model (see Appendix 1).

A simple descriptive analysis was performed of the baseline characteristics of the participants. Non-parametric testing (Wilcoxon signed-rank test) (see Appendix 2) was used to compare the difference in questionnaire responses before and after the workshop. The level of statistical significance was set at p<0.05. All statistical analysis was conducted with R Version 4.4.2 and RStudio Version 2024.09.0+375 (R Foundation for Statistical Computing, Vienna, Austria (https://www.R-project.org/)).

## Results

A total of 29 foundation doctors completed the survey, with an average age of 23.3 years, with 79% (23) of participants being between the ages of 23 and 26. Fourteen participants identified as male, while 15 identified as female, constituting 48.3% and 51.7% of the total participants, respectively, as shown in Table 1.

**Table 1:** Participant demographics. Table showing the demographics of the participants, such as age and sex, experience in laparoscopic surgery, and experience with previous surgical courses.

Demographics		% (n)
Age (in years)	23-24	34% (10)
	25-26	45% (13)
	27-28	14% (4)
	29+	7% (2)
Sex	Male	48% (14)
	Female	52% (15)
Experience		% (n)
Experience in laparoscopic surgery	None	14% (4)
	Observing	31% (9)
	Assisting	55% (16)
	Performing	0% (0)
Previous surgical courses	None	72% (21)
	Open	17% (5)
	Basic laparoscopy	14% (4)
	Advanced laparoscopy	0% (0)

Table 1 also shows the participants' previous experience in laparoscopy surgery; none of the participants have performed laparoscopic surgery. However, 55% (16) of participants reported assisting in such surgeries, and a further 31% (9) had observed laparoscopic surgeries without directly participating, while 14% (4) had no prior experience with laparoscopic surgery whatsoever. Seventy-two percent (21) of participants had no prior experience attending a basic surgical course. However, 17% (5) of participants had previously attended a basic open surgical course, and 14% (4) had participated in a basic laparoscopy surgical course. Notably, there was no one who had completed an advanced laparoscopy course (Table 1).

**Figure 2:**
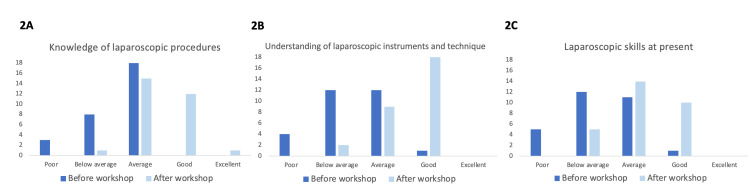
Bar charts showing the knowledge, understanding, and skills in laparoscopic surgery before and after the workshop. (A) (Left) Knowledge of laparoscopic procedures before and after the workshop. (B) (Middle) Self-perceived understanding of laparoscopic instruments and technical equipment before and after the workshop. (C) (Right) Overall laparoscopic skill before and after the workshop. Dark blue bars represent responses before the workshop and light blue bars represent responses after the workshop.

**Figure 3:**
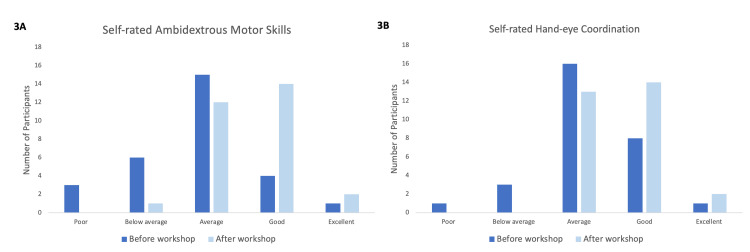
Bar charts showing self-rated ambidextrous motor skills and hand-eye coordination. (A) (Left) Self-perceived ambidextrous skills before and after the workshop. (B) (Right) Self-perceived hand-eye coordination before and after the workshop. Dark blue bars represent responses before the workshop and light blue bars represent responses after the workshop.

Self-perceived knowledge and skill set were ranked before and after the workshop on a scale of 1-5, reflecting levels ranging from poor to excellent, respectively. After the workshop, the average perceived knowledge of laparoscopic surgery increased by 37%, and the average understanding of laparoscopic instruments and technical equipment increased by 51%, as shown in Figure 2A, 2B. These changes were statistically significant with a p<0.001 for both questions. There was an average of 39% improvement in perceived overall laparoscopic skills pre- and post-course as shown in Figure 2C (p<0.001). There were also a 28% increase in perceived ambidexterity pre- and post-course and a 14% increase in perceived hand-eye coordination, as seen in Figure 3A, 3B, respectively (p<0.001 for both).

**Figure 4:**
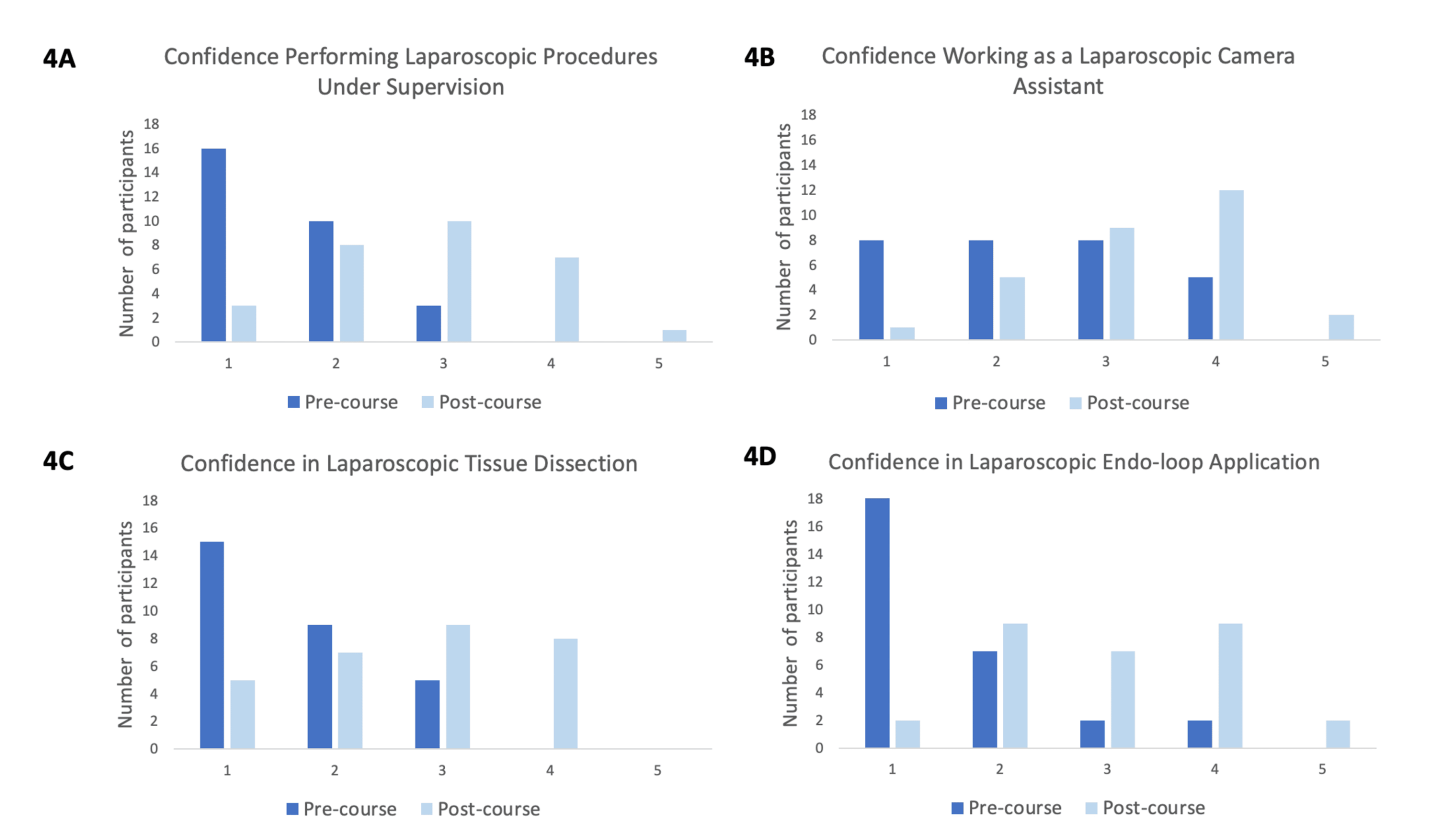
Bar charts comparing self-perceived confidence in different aspects of laparoscopic surgery before and after the course. Top row: (A) (Left) Self-perceived confidence in performing laparoscopic procedures under supervision before and after the workshop. (B) (Right) Self-perceived confidence in working as a laparoscopic camera assistant before and after the workshop. Bottom row: (C) (Left) Self-perceived confidence in laparoscopic tissue dissection before and after the workshop. (D) (Right) Self-perceived confidence in applying Endo-loop laparoscopically before and after the workshop. Dark blue bars represent responses before the workshop and light blue bars represent responses after the workshop.

Confidence in different laparoscopic skills was also ranked before and after the course on a scale of 1-5. After the workshop, the confidence in performing laparoscopic procedures under supervision increased by 82% (Figure 4A). The confidence in working as a laparoscopic camera assistant increased by 41% and confidence in laparoscopic tissue dissection and Endo-loop application increased by 63% and 89%, respectively, as shown in Figure 4B, 4C, 4D. All self-reported increases in confidence were statistically significant, with p<0.001.

Overall, when asked to rate the effectiveness of the laparoscopic appendicectomy model, 24% (7) rated it 4 out of 5 (very effective), and 76% (22) rated it 5 out of 5 (extremely effective). The model itself was easy and quick to prepare, assemble, and replicate, and it cost £0.30 ($0.40) per unit to make.

## Discussion

This survey study on a cohort of junior doctors with very little formal surgical training in laparoscopic skills showed that our inexpensive training simulation model was acceptable and effective as a form of simulation training. This model increased participants' perceived understanding of laparoscopic surgery, instruments, and techniques, and it also greatly increased overall laparoscopic skills by 51.5%. It had a smaller, yet still significant, impact on ambidexterity and hand-eye coordination (increasing it by 38.3% and 26.4%, respectively); however, these may be perceived as "innate" traits that require further practice to improve significantly. Interestingly, this workshop and model increased confidence in performing laparoscopic procedures under supervision by 115.4%, as well as increased confidence in working as a laparoscopic camera assistant, laparoscopic tissue dissection, and Endo-loop application. All of this would translate into these trainee doctors feeling more confident in laparoscopic procedures and being able to further develop their skills in theatres by getting more involved.

This modified chicken wing model is a cost-effective and easily accessible training model for early-stage laparoscopic surgery. Specifically, this simulated laparoscopic appendix model allows for the dissection and division of a simulated mesoappendix using only laparoscopic scissors and forceps, providing realistic tactile feedback, with the tissue closely mimicking that of human tissue, enhancing the realism of the simulation compared to silicone models. It validates a modified version of O'Connor and Paraoan's model [7] in a small cohort and showed that trainees found this model to be both acceptable and helpful in developing their laparoscopic skills and knowledge, as well as in boosting their confidence to participate in laparoscopic surgeries. It is also straightforward to prepare and replicate, ensuring consistent simulated training experiences which can be done with simple equipment, without having to deal with the ethical or logistical cost of cadaveric or live animal models. We omitted the use of electrocautery as this added more expenses and required multiple electrosurgical generators.

Simulation training in surgery is a valuable, yet underutilized, tool for building confidence among medical students and junior doctors [2]. By providing a controlled, risk-free environment, it allows doctors to practice and refine their skills without the pressure of complications and time pressure of a busy operating schedule. Having more hands-on practice will improve their confidence and skill level in a quicker period, as in theatres trainees will only get limited time to practice skills or be taught specific techniques. This method of training is particularly beneficial in laparoscopy surgery, where precise hand-eye coordination and navigating a 3D space using a 2D image take practice to get used to, as does building familiarity with laparoscopic tools and instruments. The significant improvements observed in participants' perceived knowledge and skills, as well as their increased confidence in performing various laparoscopic tasks, highlight the effectiveness of simulation training, which is evidenced by Zendejas et al.'s meta-analysis which highlights the benefits of simulation training for laparoscopic surgery [8].

As trainee doctors feel more confident in their abilities, they are likely to be more proactive and engaged in operating theatres, taking on more responsibilities and further developing their surgical competencies. Lees et al. suggest that internal factors such as operative experience, self-perception, and individual skill development in surgical trainees improve surgical confidence and hence enhance their learning experience but also contribute to them becoming well-trained and more independent surgeons, improving patient outcomes [9].

With the shift to laparoscopic and robotic surgery, it is important to incorporate minimally invasive surgical training from an earlier stage. This is the first study to our knowledge to trial and validate an inexpensive and accessible training model for simulated laparoscopic appendicectomy in a small cohort. However, our study's limitations include the small cohort size and the outcomes being prone to self-report biases, including social desirability bias and response bias, due to the subjective nature of self-assessment questionnaires. Going forward, this model needs to be trialed in larger groups to increase its power and with objective and measurable outcomes to produce more robust evidence. Another potential limitation of this model is the potential costs of supervisors; we found that by organizing an official teaching day accredited by the local deanery, surgical registrars and consultants volunteered their time to be supervisors, eliminating the cost.

## Conclusions

This simulated laparoscopic appendix model is inexpensive and easy to prepare and assemble, hence reducing the barriers to high-fidelity simulation for laparoscopic surgical training. Trainees found the model acceptable and helpful in developing their laparoscopic skills and knowledge as well as increasing their confidence in participating in laparoscopic surgery. This simulation model is a practical, relatively inexpensive, and effective training model which can be used for early-stage laparoscopic training for medical students and junior doctors, enabling them to get the most out of surgical placements and time in theatres. 

## Appendices

Appendix 1

NHS East of England Surgical Hub Day: Pre-workshop Questionnaire

1.     How old are you?

a.     Under 23

b.     23-24

c.     25-26

d.     27-28

e.     29 and over

2.     Which gender do you identify as:

a.     Male

b.     Female

c.     Other

d.     Prefer not to say

3.     How much previous experience do you have in laparoscopic surgery?

a.     None

b.     Observing

c.     Assisting

d.     Performing

4.     Have you previously attended surgical skills courses?

a.     None

b.     Open surgical skills

c.     Basic laparoscopic surgical skills

d.     Advanced laparoscopic surgical skills

5.     Please rate your knowledge of laparoscopic procedures

a.     Poor 

b.     Below average

c.     Average

d.     Good

e.     Excellent

6.     Please rate your understanding of laparoscopic instruments and techniques

a.     Poor 

b.     Below average

c.     Average

d.     Good

e.     Excellent

7.     Please rate your level of laparoscopic skills currently

a.     Poor 

b.     Below average

c.     Average

d.     Good

e.     Excellent

8.     Please rate your level of ambidextrous motor skills

a.     Poor 

b.     Below average

c.     Average

d.     Good

e.     Excellent

9.     Please rate your level of hand-eye coordination

a.     Poor 

b.     Below average

c.     Average

d.     Good

e.     Excellent

10.  Please rate your confidence in performing laparoscopic procedures under supervision 

1                      2                      3                      4                      5

11.  Please rate your confidence in working as a laparoscopic camera assistant 

1                      2                      3                      4                      5

12.  Please rate your confidence in dissecting tissue laparoscopically

1                        2                      3                      4                      5

13.  Please rate your confidence in applying the Endo-loop laparoscopically                              

 1                      2                      3                      4                        5

NHS East of England Surgical Hub Day: Post-workshop Questionnaire

1.     Please rate your knowledge of laparoscopic procedures after the workshop 

a.     Poor 

b.     Below average

c.     Average

d.     Good

e.     Excellent

2.     Please rate your understanding of laparoscopic instruments and techniques after the workshop

a.     Poor 

b.     Below average

c.     Average

d.     Good

e.     Excellent

3.     Please rate your level of laparoscopic skills after the workshop 

a.     Poor 

b.     Below average

c.     Average

d.     Good

e.     Excellent

4.     Please rate your level of ambidextrous motor skills after the workshop

a.     Poor 

b.     Below average

c.     Average

d.     Good

e.     Excellent

5.     Please rate your level of hand-eye coordination after the workshop

a.     Poor 

b.     Below average

c.     Average

d.     Good

e.     Excellent

6.     Please rate your confidence in performing laparoscopic procedures under supervision after the workshop

1                      2                      3                      4                      5

7.     Please rate your confidence in working as a laparoscopic camera assistant after the workshop

1                      2                      3                      4                      5

8.     Please rate your confidence in dissecting tissue laparoscopically after the workshop

1                      2                      3                      4                      5

9.     Please rate your confidence in applying the Endo-loop laparoscopically after the workshop

 1                      2                      3                      4                      5

10.    Please rate the effectiveness of this laparoscopic appendicectomy model

 1                      2                      3                      4                      5

Appendix 2

**Table 2:** Percentage change between pre- and post-questionnaire and p-values from the Wilcoxon signed rank test.

Question	Percentage change (%)	P-value	Significance
Laparoscopic knowledge (2A)	37	3.26e-06	p<0.001
Laparoscopic instruments (2B)	51	1.116e-06	p<0.001
Laparoscopic skills (2C)	39	3.686e-07	p<0.001
Ambidexterity (3A)	28	1.115e-05	p<0.001
Hand-eye coordination (3B)	14	0.0009212	p<0.001
Confidence in performing laparoscopic surgery (4A)	82	4.403e-06	p<0.001
Confidence in assisting with a laparoscopic camera (4B)	41	1.957e-06	p<0.001
Confidence in laparoscopic tissue dissection (4C)	63	6.525e-06	p<0.001
Confidence in laparoscopic Endo-loop application (4C)	89	3.03e-06	p<0.001
